# Inspection of Reactor Steel Degradation by Magnetic Adaptive Testing

**DOI:** 10.3390/ma12060963

**Published:** 2019-03-22

**Authors:** Gábor Vértesy, Antal Gasparics, Ildikó Szenthe, Ferenc Gillemot, Inge Uytdenhouwen

**Affiliations:** 1 Hungarian Academy of Sciences, Centre for Energy Research, 1121 Budapest, Hungary; vertesy.gabor@energia.mta.hu (A.G.); szenthe.ildiko@energia.mta.hu (I.S.); gillemot.ferenc@energia.mta.hu (F.G.); 2 SCK•CEN Belgian Nuclear Research Centre, 2400 Mol, Belgium; inge.uytdenhouwen@sckcen.be

**Keywords:** steel degradation, nuclear reactor pressure vessel, magnetic nondestructive evaluation, magnetic adaptive testing

## Abstract

Degradation of nuclear pressure vessel steel materials, 15Kh2NMFA type and A508 Cl2 type (definition is given in the text) were investigated by a novel magnetic nondestructive testing method, so-called Magnetic Adaptive Testing (MAT), which is based on systematic measurement and evaluation of minor magnetic hysteresis loops. The measured samples were thermally treated by a special step cooling procedure, which generated structural changes in the material. It was found that this type of degradation can be easily followed by magnetic measurements. Charpy impact test were also performed and the results were compared with the magnetic parameters. In case of 15Kh2NMFA steel, a good, reliable and closely linear correlation was found between magnetic descriptors and transition temperature.

## 1. Introduction

Pressurized water reactors are typical in most of the currently operating nuclear power plants. The reactor pressure vessel (RPV) is one of the most important safety related part of these plants, and determines the lifetime of them. It is operated at elevated temperature, high pressure, neutron radiation, thermal ageing and low cycle fatigue, which are the main environmental effects that degrade the RPV material properties during service. Safe operation rules require that the material properties never degrade below the safety code requirements. When a nuclear reactor starts to operate, its pressure vessel steel is still not degraded. During its whole operation time, the actual quality of the pressure vessel should be regularly checked. The RPV walls are low alloyed ferromagnetic CrNiMo or CrMoV steels. Their critical parts are the forged rings and their welds at the core level. 

The material properties of the reactor pressure vessels are periodically monitored by so-called surveillance programs. In the surveillance program, many samples are used, which are placed inside the reactor from the start of operation, and exposed to the same (or even a slightly larger) neutron fluence and thermal conditions as the RPV walls.

The surveillance specimen sets generally include tensile, Charpy impact and fracture toughness testing samples; however, the latter ones are missing in some old units. The impact and fracture mechanical specimens are typically 55 x 10 x 10 mm size blocks. According to the design and authority requirements, specimen sets are periodically withdrawn from the RPV and tested. All mechanical tests are destructive. One of the testing is the well-known destructive Charpy impact method. Charpy impact tests were started more than one hundred years ago [1] to use as a regular test method of material toughness. A swinging hammer breaks the (standard size and shape) sample to be tested, and the energy absorbed by the fracture of the specimen is a characteristic property of the material. The absorbed energy highly depends on the testing temperature. The temperature where the absorbed energy is 41 J is called the ductile-brittle transition temperature (TTKV_41_). At least a dozen of surveillance Charpy samples are used for one single TTKV evaluation. The Charpy tests are conventional mechanical tests. Nowadays, other tests are also used for direct measurement of the toughness of the RPV materials. The surveillance samples during irradiation become highly radioactive, testing them is available only in hot laboratories and it is very costly.

The original surveillance sets are planned for the design lifetime. Generally, the RPV’s real safe lifetime is much longer than the design lifetime, and for this extended operational lifetime, new specimen sets should be designed, produced and located in the surveillance channels. Instead of destructive tests, application of nondestructive tests would be rather helpful.

However, nondestructive tests in contrast to the destructive ones, do not determine directly the material characteristics. Nondestructive tests, before any real practical application, should be strictly compared to the standardized destructive ones in a long series of careful inspections. This is why until now nondestructive tests were not able to replace the destructive tests. To solve this problem, a sound foundation for verification of the needed correlation is required. Several nondestructive methods were recommended and presently tested. Magnetic methods, and among them mainly hysteresis methods, play a special role, because a close analogy exists between modification of the steel microstructure if a mechanical load is applied, when movement of dislocations happens, and under an applied magnetizing field, when movement of magnetic domain walls happens. In ferromagnetic materials movement of dislocations and movement of the domain walls sense micro-structural imperfections on their way through the material. This close correlation between mechanical and magnetic hardness is well interpreted. Usually magnetic methods are simple and not expensive, and the electronic control unit can be put outside the protected area when the radioactive samples are measured.

Numerous experiments were described, which proved technical applicability of magnetic ways for quantitative indication of the degradation of steels, due to micro-structural modifications, which cause embrittlement. Cold rolling [2], thermal treatment [3] and fast neutron irradiation effect on RPV steels and welds were tested, see e.g., [4,5,6,7,8,9,10,11]. Materials, both as received and irradiated ones, with different fluences, were studied.

One promising candidate technics applied to determine steel degradation is the method of Magnetic Adaptive Testing (MAT). This method is based on detection of minor magnetic hysteresis loops. This is a multi-parametric, sensous and powerful procedure of magnetic inspection introduced recently, see [12,13]. In a previous work [14], samples made of JRQ, 15CH2MFA and 10ChMFT type steel material were investigated by MAT. A533 type B Class 1 steel known as **JRQ** (a special type of heat-treated steel with increased impurity, optimized for investigation of correlation between radiation embrittlement and other physical properties), and the base (**15CH2MFA**) and welding (**10ChMFT**) material from the WWER 440-type Russian reactor.

The samples were irradiated by E > 1 MeV energy fast neutrons with total neutron fluence of 1.58 x 10^19^–11.9 x 10^19^ n/cm^2^. Monotonous correlation was experienced between MAT degradation parameters and the neutron fluence in all three types of the steels.

In this work, applicability of MAT is shown for the detection of the degradation of the reactor steel, caused by thermal treatment. The research work was done in the frame of the NOMAD project (see acknowledgement). This investigation serves as a first step of the project; later, different samples will be irradiated by neutrons and will be measured by MAT and also by other methods. The final goal is to find the most appropriate method, which can be applied in practical applications.

## 2. Sample Preparation and Processing 

Three Charpy specimens from 15Kh2NMFA material (Russian standard steel for WWER/1000 reactors used as reference steel from the Joint Research Center Institute for Energy, Petten, The Netherlands ) and three Charpy specimens from A508 Cl2 material (standard steel according to the ASTM A508 standard) were measured. The chemical compositions of samples are given in Table 1 and Table 2. 

The samples were aged by thermal treatments. In the literature, several forms of step-cooling technology can be found. Most of them are used for accelerated evaluation of thermal ageing sensitivity. The main consideration at the selection of the thermal treatment was to produce microstructural changes similar to those that occur at irradiation. Of course to produce the same microstructure as the result of irradiation is impossible by any other ageing technology. Quenching and annealing (selected for this purpose) may result in similar hardness as irradiation, but with a very different microstructure. To avoid the rough change in grain sizes and microstructure the applied treatment temperature should be below the annealing temperature (660 °C for A508 and 640–655 °C for 15Kh2NMFA).

The effectiveness of the step cooling can be expected by the so-called step cooling sensitivity formula based on the chemical composition:

J = (Mn + Si) × (P + Sn) × 10^4^ (1)

For 15Kh2NMFA, material Equation (1) reads as:

(0.42 + 0.29) × (0.012 + 0.003) × 10^4^ = 106.5 (2)

and for 508 material, Equation (1) reads as:

(0.61 + 0.26) × (0.005 + 0.001) × 10^4^ = 52.2 (3)

A higher J value means a higher transition temperature shift as a result of the step cooling. If the J value is near or around to the value of 100, the step cooling will result in a considerable shift. Moreover, the shift depends on the chemical composition, pollution and previous heat treatment history. The following factors are taken into consideration at the selection of the step cooling technology: the maximum temperature of the step cooling should be below the annealing temperature to avoid rough changes in the microstructure, the step cooling technology should produce two differently aged samples; however, few examples can be found in the literature for two phase step cooling of steels. 

A step cooling technology elaborated for the 15Kh2NMFA steels and meets the above mentioned requirements is known and published [15]. For the A508 steel no such technology is available, and the above calculated sensitivity is much less than the sensitivity of the 15kh2NMFA steel. Nevertheless, the same technology was used for both steels, to shorten the specimen preparation period. 

During thermal treatment the surface is oxidizing and a thin layer of cinders is created. To avoid the disturbance of the magnetic techniques the material has been thermally treated in blocks and the specimens had been cut after the treatment.

Two types of step cooling were performed. Two samples of each series were processed by “Thermal treatment 1”, two other pieces were processed by “Thermal treatment 2” and the third pieces of each series were not heat treated; they served as reference samples. 

**Figure 1:**
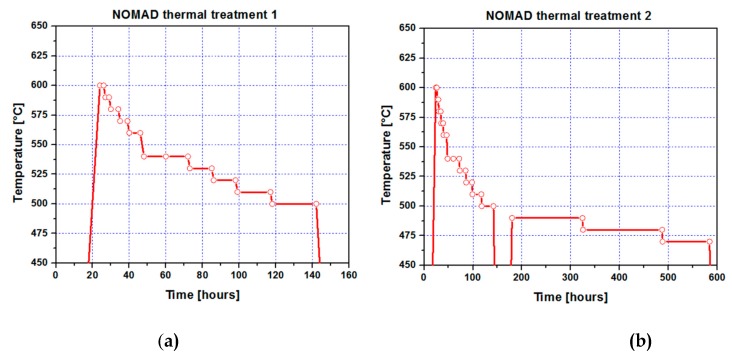
(**a**) Step cooling ”thermal treatment 1”; (**b**) step cooling ”thermal treatment 2”.

In thermal treatment 1, slow heating was applied up to 600 °C, then 10–20 °C step cooling down to 500 °C. The periods of intervals spent on stable temperature at each step are given as follows: 600 °C /2 h + 590 °C /2 h + 580 °C /4 h + 570 °C /4 h + 560 °C /6 h + 540 °C /12 h + 530 °C /12 h + 520 °C /12 h + 510 °C /18 h + 500 °C /24 h (see Figure 1a). 

In thermal treatment 2, slow heating was applied up to 600 °C, then 10–20 °C step cooling down to 500 °C. The periods of intervals spent on stable temperature at each steps are given as follows: 600 °C /2 h + 590 °C /2 h + 580 °C /4 h + 570 °C /4 h + 560 °C /6 h + 540 °C /12 h +530 °C /12 h + 520 °C /12 h + 510 °C /18 h + 500 °C /24 h + 490 °C /144 h + 480 °C /162 h +470 °C /96 h (See Figure 1b).

## 3. Results

### 3.1. Charpy Impact Testing

Our final purpose was to compare the magnetic parameters measured in a non-destructive way with the Charpy impact testing shifts on irradiated samples. Since irradiated samples are radioactive and can be tested only in hot laboratories, these thermally aged samples were used to develop the testing technology. The following testing methods were selected to measure the ageing shift, and the homogeneity of the materials: ASTM-23-16b for Charpy impact testing [16], ASTM 1921-18 for fracture toughness testing [17] and ASTM 92-17 for hardness testing [18].

Charpy impact testing used to determine the 41 Joule transition temperature. 300 J MFL PMT PSW300M type impact tester (Maschinenfabrik Liezen und Gießerei Ges.m.b.H. Werkstraße 5, 8940 Liezen, Austria) was used for the testing with ISO tup. The machine was calibrated and authenticated by an authorized company. The specimens were cooled to the testing temperature in a vessel containing liquid n-pentan. The cooling of n-pentan was made by low temperature nitrogen vapor circulated in cooling pipes. An electronic temperature adjustment device controlled the flow of nitrogen vapor. The n-pentan temperature is measured by K-type thermocouples brazed into a Charpy size steel block. Another K type thermocouple with a hand data logger was used to check the validity of the temperature. The specimens cooled for 15–45 minutes according the required testing temperature (at deeper temperatures, a longer cooling time was selected). An electric motor was used to mix and homogenize the n-pentane to avoid temperature gradient. The specimens had been overcooled with 1–3 °C to compensate the heating during the manipulating time from the liquid to the anvil and the time required for the hammer to reach the specimen. The manipulation time was less than 3 seconds. The absorbed energy during the fracture of the specimens was recorded. Pictures of the fracture surface of the broken specimens were made and evaluated. In the few cases of slag inclusions found in the specimens, the specimens were censored. 

After this censoring, a tangent hyperbolic curve was fit on the remaining data points and 41 Joule criteria evaluated. According to the standards and the international practice artificial absorbed energy values at −200 and −150 °C temperature were used to enhance the fitting. Lower bound of the Charpy results for all low alloyed steels was between 3–6 Joules. Moreover, 95% probability curves fit on the measured data to evaluate the scatter. The whole testing procedure was made according to the ASTM 23-11 standard. 

**Figure 2:**
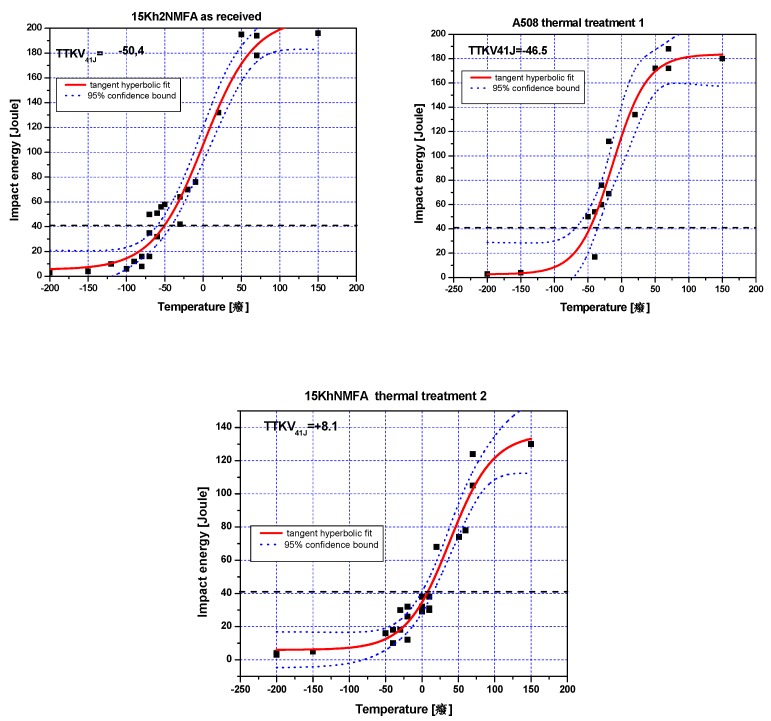
Results of the Charpy impact testing and evaluation of the transition temperature (41J criteria) on 15Kh2NMFA samples as received, and thermally treated.

The data, the tangent hyperbolic fittings and the scatter bounds are shown in Figure 2 for 15Kh2NMFA samples. In case of A508 Cl2 samples the same procedure was performed (not shown here). The results are summarized in Table 3.

It can be seen that this thermal treatment caused a regular modification in the structure of 15Kh2NMFA material and a monotonous increase of transition temperature was experienced due to the treatment. On the other hand, the same treatment was not successful for the modification of transition temperature in case of the other (A508 Cl2 type) material. The most probable explanation is that this step cooling technology was elaborated for the 15Kh2NMFA steels, and to achieve similar result in mechanical characteristics, A508 material requires other thermal treatment with different parameters.

### 3.2. Magnetic Adaptive Testing

**Figure 3:**
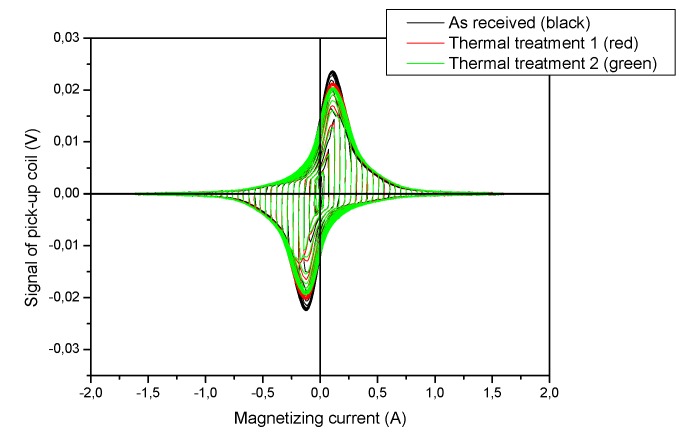
Measured permeability loops of the three (as received and thermally treated) 15Kh2NMFA samples.

Magnetic adaptive testing systematically measures series of magnetic minor hysteresis loops, starting from the demagnetized state up to closely saturated state. The amplitude of magnetizing field was increased by steps from loop to loop. All samples in the investigated sample sets were measured by the same parameters. As shown in the description of the theoretical Preisach hysteresis model [19], these complex sets of minor hysteresis loops contained a lot of information about the material behavior. Measuring equipment, called Permeameter (Institite of Physics, Prague, Czech Republic), was designed and built for the purpose of MAT measurements. In the experiment—by applying a magnetizing yoke, put on the surface of the sample, which magnetized the specimen—the differential magnetic permeability was measured and then evaluated. The size of the sample determines the size of the magnetizing yoke, which was C-shaped laminated Fe-Si transformer core. In our measurement, the cross-section of the yoke was 10 mm x 5 mm, the total outside length was 18 mm and the height of the yoke was 22 mm. Magnetization was made by a magnetizing current, led into the 200 turns magnetizing coil, wound on the bow of the yoke. Voltage output signal was detected by a 75 turns pick-up coil, wound around the yoke leg. A triangular waveform magnetizing current was applied. The slope of the current (time variation) was fixed and its amplitude was increased step by step. The output signal is proportional to the differential permeability if the magnetizing current increases linearly with time. As an illustration, the measured permeability loops are presented in Figure 3 for 15Kh2NMFA steel samples. The sets of minor loops with step-by-step increasing amplitudes are clearly seen. 

**Figure 4:**
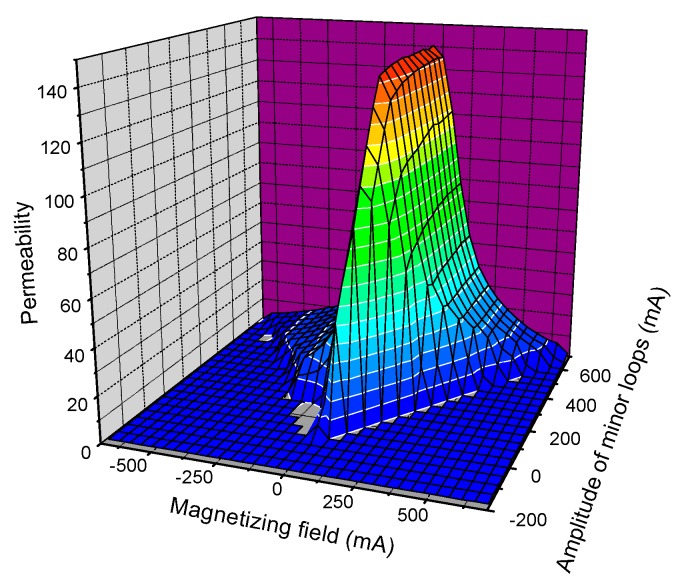
Permeability matrix of the as received 15Kh2NMFA sample.

The whole measurement is controlled by a Personal Computer (PC, Acer, Nalganga Dam, Taiwan). For each minor loop, a data file was generated, which was then evaluated by a program. After filtering and interpolating these measured data, a *μ* ≡ *μ*(*h_a_*,*h_b_*) matrix was generated, the coordinates of which represent the actual magnetic field value, *h_a_*, on the actual minor loop with amplitude *h_b_*. These *μ* matrix-elements were named “MAT-descriptors”, which characterize the structure variation of the studied sample. As an illustration, the calculated permeability matrix is presented in Figure 4 for the as received 15Kh2NMFA sample.

Another evaluation program was also developed, which divided the actual matrix elements by the corresponding matrix elements of the reference sample, in such a way generating a standardized *μ*(*x*) matrix, the elements of which (*μ*(*x*)-degradation functions) contain all of the information about material degradation. Here, *x* is the independent parameter. The parameters in our work were the total time of the step cooling on one hand, and the transition temperature on the other, determined independently in the samples as shown above. The detailed description of the whole MAT procedure is in [13].

In the case of the measurements, presented here, an open magnetic circuit was formed by the magnetizing yoke, which was put on the surface of the flat sample, and by the sample itself. It is perfectly closed, because an air gap always exists between the yoke and sample. It means that some magnetic flux is scattered, consequently exact value of the magnetic field inside the specimen is not known in the applied experimental arrangement. Due to this fact, the magnetizing current was used as *h_a_* and *h_b_* values when the *μ* ≡ *μ*(*h_a_*,*h_b_*) matrix elements are calculated, instead of the magnetic field. 

**Figure 5:**
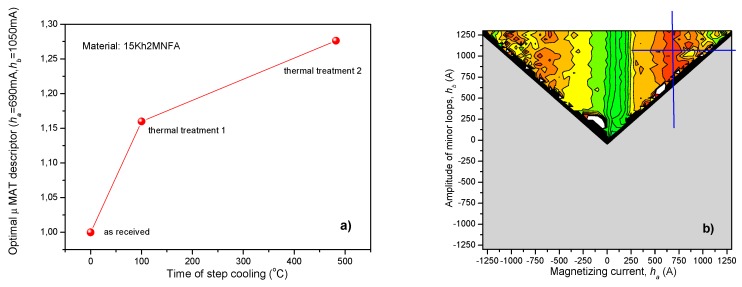
(**a**) Optimally chosen normalized Magnetic Adaptive Testing (MAT) descriptor as a function of the time of step cooling for 15Kh2NMFA material; (**b**) the corresponding map of sensitivity, which shows the position of this descriptor.

**Figure 6:**
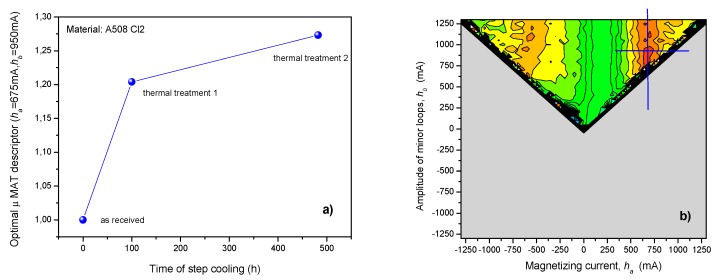
(**a**) Optimally chosen normalized MAT descriptor as a function of the time of step cooling for A508 Cl2 material; (**b**) the corresponding map of sensitivity, which shows the position of this descriptor.

**Figure 7:**
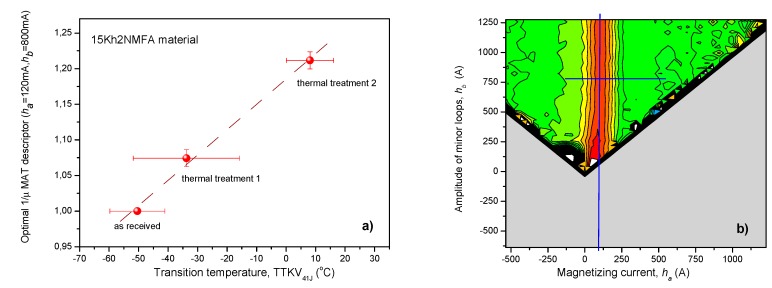
(**a**) Optimally chosen normalized MAT descriptor as a function of transition temperature for 15Kh2NMFA material; (**b**) the corresponding map of sensitivity, which shows the position of this descriptor.

The calculated *μ*(*x*) matrix contains a great number of elements. The task is to choose the most sensitive and at the same time reliable enough element (MAT descriptor), which characterizes the actual degradation of the material. This choice can be done by using another matrix, the so-called sensitivity matrix. The sensitivity of each degradation function is calculated with respect to the independent variable. The obtained “sensitivity map” shows the sensitivity in the plane of the field coordinates (*h_a_*,*h_b_*). The sensitivity of each degradation function is calculated as the slope of its linear regression. In the sensitivity map figure, this slope is shown as a shade of color. For example, as can be seen below in Figure 5a, Figure 6a and Figure 7a, a red color (top of the “hills”) expresses the largest sensitivity of the actual MAT descriptor with respect to the time of heat treatment and/or transition temperature. This map also shows the reliability of the magnetic descriptor: large plateaus are favorable, because in these areas, the MAT descriptor depends only very limitedly on the exact choice of the field coordinate values *h_a_* and *h_b_*. This is the way optimal descriptors should be chosen. 

MAT measurements were performed on the two series of the above described reactor steel samples after step cooling. As a first step of evaluation, MAT descriptors were considered as functions of the time of step cooling. Figure 5 shows the result, obtained on the 15Kh2NMFA samples. In Figure 5a the optimally chosen MAT descriptor can be seen as a function of the time of step cooling. The optimally chosen descriptor is characterized by μ(*h_a_* = 690 mA, *h_b_* = 1050 mA) values. The corresponding sensitivity map is shown in Figure 5b. The sensitivity map indicates that the most sensitive (red) area is rather large, which ensures the good reproducibility and reliability of the MAT descriptor. The (blue) lines in the sensitivity map, crossing each other, show the position of the μ(*h_a_* = 690 mA, *h_b_* = 1050 mA) descriptor.

Figure 6 shows the result, obtained on the A508 Cl2 samples. Again, in Figure 6a, the optimally chosen MAT descriptor can be seen as a function of the time of step cooling. In this case, the optimally chosen descriptor is characterized by μ(*h_a_* = 675 mA, *h_b_* = 950 mA) values, which is very close to the descriptor, found as the best in the previous case (15Kh2NMFA material). The slight difference can be attributed to the different material. The corresponding sensitivity map is shown in Figure 6b, which proves in also these cases also the high reproducibility of the measurement.

As it is seen in the graphs, regular, monotonously increasing correlation was found between the optimally chosen MAT degradation function and the time of step cooling. The magnetic parameters, due to 142 hours annealing, were modified by about 16% compared to the reference sample in case of 15Kh2NMFA material and by about 21% in case of A508 Cl2 material. The second, 482 hours heat treatment caused additional changes in the structure of the material, the magnetic parameters, were modified by about 30% compared to the reference sample in case of 15Kh2NMFA material and by about 27% in case of A508 Cl2 material. 

As the second step of evaluation, MAT descriptors were considered as functions of the transition temperature. We would like to emphasize that one single measurement (measurement of series of minor permeability loops) was performed on the samples; however, during MAT evaluation, the generated big data pool was considered and those descriptors were chosen that resulted in the best correlation with the independent parameter. In this second case, the independent parameter is the transition temperature. The result for 15Kh2NMFA material is shown in Figure 7. In this case, the optimal descriptor was 1/μ(*h_a_* = 120 mA, *h_b_* = 800 mA). The sensitivity map is also presented. As can be clearly seen, this descriptor is taken from a different area of the sensitivity map than in the case of MAT parameters versus time of step cooling. The good reproducibility is proven in this case, too. 

The correlation between MAT parameters and transition temperature seems to be very close to a linear one. Because of this, a linear fit was done on the points and shown in Figure 7a. We are aware that any fit on three existing points is usually not justified; thus, this line, which connects the points, is considered only as for driving the eye. However, the monotonous (at least closely linear) correlation is evident. 

**Figure 8:**
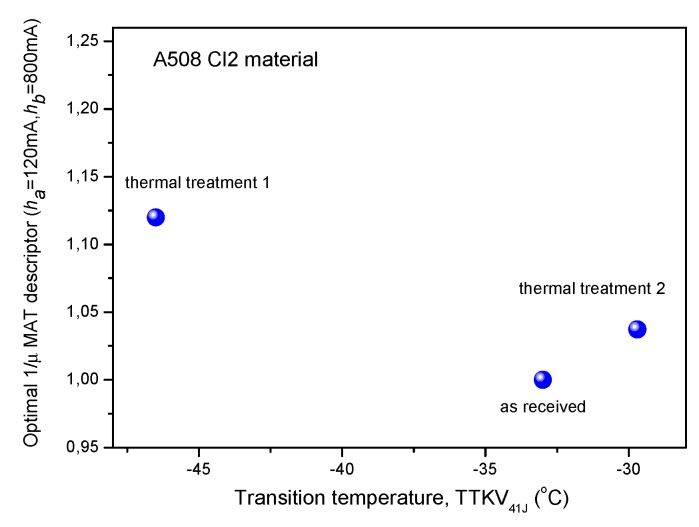
Optimally chosen normalized MAT descriptor as a function of transition temperature for A508 Cl2 material.

Figure 8 shows the same MAT descriptor for A508 Cl2 material (as a function of transition temperature), which was found the best in case of 15Kh2NMFA material. It is evident that no regular correlation exists in this case between MAT parameter and transition temperature. It is believed that this irregular correlation comes from the inappropriate step cooling.

## 4. Discussion

It was shown that the method of Magnetic Adaptive Testing was found to be a sensitive and reliable way to characterize the structural changes in reactor pressure vessel material. A definite and monotonous correlation was found between the time of step cooling process and MAT descriptors for both types of the investigated materials. The structural changes of reactor pressure vessel steels, caused by heat treatment were investigated and discussed in several papers. Both optical microscopy and scanning electron microscopy (SEM) were performed. It was found that visible changes in material structure are generated by this processing [20,21,22].

The modification of the transition temperature due to this thermal treatment can also be followed by this nondestructive method, and a closely linear correlation was found between MAT descriptors and Charpy 41 Joule transition temperature. 

It is interesting and worth of mentioning that by this magnetic measurement, we were able to determine different magnetic descriptors. One of them, characterized by μ (*h_a_* = 700 mA), *h_b_* = 1000 mA) values) is sensitive to the structural changes, generated by thermal treatment, while the other one (characterized by 1/μ (*h_a_* = 120 mA, *h_b_* = 800 mA) values) is sensitive to the modification of transition temperature. 

This result indicates an important useful property of Magnetic Adaptive Testing, namely its multi-parametrical character: from the big data pool, which is generated by the method, those parameters can be chosen, which characterize the best the actual degradation of the material. This feature was analyzed in another work [23]. In general those magnetic descriptors should be used for calibration, which are monotonous and most sensitive to the investigated material degradation.

Magnetic adaptive test can be applied to inspection of all ferromagnetic structural material practically without limitation. The surface should be flat and good enough (or at least similarly rough for all samples within the investigation series) and measurement parameters (slope of magnetizing current, step of amplitude increase, frequency of sampling, etc.) should be kept rigorously the same within one series of measurement. Evidently magnetic descriptors, characterized by different μ(*h_a_*,*h_b_)* values are optimal for different materials. 

## 5. Conclusions

It was shown that the MAT method is suitable for nondestructive structural inspection of reactor pressure vessel steel: optimally chosen degradation functions characterize structural changes of this material reliably and parameters can be determined easily. 

A thermal treatment was applied, which generated structural changes in the material. It was found that this type of degradation can be determined by magnetic measurements. Charpy impact test were also performed and the results were compared with the magnetic parameters. In case of 15Kh2NMFA steel, a good, reliable and closely linear correlation was found between magnetic descriptors and transition temperature. In the case of other material (A508 Cl2) the heat treatment procedure, although it caused modification in material structure, was not effective enough to produce measurable change in the transition temperature.

In conventional magnetic nondestructive measurement methods, based on hsyteresis measurement, the samples should be magnetically saturated: the major hysteresis loop is measured. It is a significant drawback, because in the realistic cases, an air gap always exists between the sample and the magnetizing yoke, which means that the real saturation is practically not possible. In MAT the magnetic saturation is not necessary, information is taken from minor loops. It also means that the size of magnetizing yoke can be rather small. MAT does not result absolute values of magnetic parameters, it only compares the magnetic parameters of the samples to be measured in the same series with similar parameters of measurement. Nevertheless, this method gives a powerful tool to compare samples and to obtain information about the material degradation.

If we want to apply MAT in practice, we should start the work on a series of samples with known material degradation (determined by standard destructive tests), after which, in the knowledge of this reference curve, any unknown sample can be successfully characterized. 

## Figures and Tables

**Table 1:** Chemical composition of 15Kh2NMFA material.

C%	Mn%	Si%	S%	P%	Cr%	Ni%	Mo%	V%	Cu%	Co%	Sb%	Sn	As%
0.16	0.42	0.29	0.08	0.012	1.97	1.29	0.52	0.12	0.12	0.06	0.001	0.003	0.003

**Table 2:** Chemical composition of A508 Cl2 material.

C%	Mn%	Si%	S%	P%	Cr%	Ni%	Mo%	V%	Cu%	Co%
0.25	0.61	0.26	0.008	0.05	0.37	0.7	0.63	0.01	0.06	0.02

**Table 3:** Results of Charpy testing of step cooled steel specimens.

Material	TTKV 41J [°C] (As Received)	TTKV 41J [°C] (Thermal Treatment 1)	TTKV 41J [°C] (Thermal Treatment 2)
A508 Cl2	−33 ± 14	−46.5 ± 11	−29.7 ± 9
15Kh2NMFA	−50.4 ± 9.3	−33.8 ± 18	+8.1 ± 8
